# Multiple Isoforms of Anti-Lipopolysaccharide Factors and Their Antimicrobial Functions in the Ridgetail Prawn *Exopalaemon carinicauda*

**DOI:** 10.3390/md16050145

**Published:** 2018-04-27

**Authors:** Xinjia Lv, Shihao Li, Chengsong Zhang, Jianhai Xiang, Fuhua Li

**Affiliations:** 1 CAS Key Laboratory of Experimental Marine Biology, Institute of Oceanology, Chinese Academy of Sciences, Qingdao 266071, China; xjlubio@163.com (X.L.); chs-zhang@163.com (C.Z.); jhxiang@qdio.ac.cn (J.X.); 2 Laboratory for Marine Biology and Biotechnology, Qingdao National Laboratory for Marine Science and Technology, Qingdao 266237, China; 3 University of Chinese Academy of Sciences, Beijing 100049, China

**Keywords:** anti-lipopolysaccharide factor, antimicrobial, RNA interference, flora balance, crustacean

## Abstract

As a kind of antimicrobial peptides (AMP) in crustacean, anti-lipopolysaccharide factors (ALFs) have broad spectrum antimicrobial activities. In the present study, we identified four ALF genes, *EcALF2-5*, from the ridgetail prawn *Exopalaemon carinicauda*. Tissue distribution analysis showed that *EcALF2* and *EcALF4* transcripts were mainly located in gill, epidermis, and stomach, while *EcALF3* and *EcALF5* were mainly in hemocytes. Peptides corresponding to the LPS binding domain (LBD) of EcALFs were synthesized for analyzing their antimicrobial activities. Minimal inhibitory concentration (MIC) analysis showed that the synthetic LBD peptides of EcALF3 and EcALF4 could inhibit the growth of Gram-positive and Gram-negative bacteria, while the synthetic LBD peptides of EcALF2 and EcALF5 showed antibacterial activity against *Vibrio*. Incubation of white spot syndrome virus (WSSV) with the synthetic LBD peptides of EcALF3, EcALF4, and EcALF5 could reduce the in vivo viral copy number in WSSV-infected prawns. After silencing of *EcALFs*, *Vibrio* exhibited a rapid proliferation in the hepatopancreas of the prawn. The present data showed the important function of different *EcALFs* in modulating the in vivo bacterial and viral propagation in *E. carinicauda*. This study will provide new clues into the disease control in aquaculture.

## 1. Introduction

Antimicrobial peptides (AMPs) are a kind of immune effectors in the innate immunity of the living organisms. Most AMPs are small proteins with 10–100 amino acid residues which show certain activities against bacteria, fungi, or viruses. Since the first AMP named cecropin was isolated from the pupae of the cecropia moth *Hyalophora cecropia* [1], thousands of AMPs have been found in various species to date, such as defensins and cathelicidins in mammals [2], thionins and lipid transfer proteins (LTPs) in plants [3], attacins and lebocins in insects [4]. In crustaceans, about 15 kinds of AMPs have been reported, including the most common AMPs penaeidins, crustins, and anti-lipopolysaccharide factors (ALFs) [5]. In the past decade, ALFs have received more and more attentions for their broad-spectrum activities against bacteria and viruses. 

ALFs consist of more than 100 amino acid residues with a signal peptide at the N-terminal, and their secondary structure usually contains several alpha helixes and beta sheets [6]. The LPS binding domain (LBD), as the functional domain of ALF, contains two cysteine residues that can form a disulfide bond to stabilize the beta sheet structure of the domain [7,8,9]. Since the first ALF was identified from hemocytes of the horseshoe crab *Limulus polyphemus*, its LPS binding activity and antibacterial characters have been confirmed [10,11]. Then, its crystal structure was then elucidated [7,12]. Up to now, a number of ALFs have been reported in various crustacean species such as shrimp, lobster, crayfish, and crab [13,14,15,16]. The expression level of ALF could be upregulated when animals were stimulated by bacteria or viruses [17,18]. Both synthetic peptides of LBD and recombinant ALF proteins exhibited certain antibacterial activities [16] or inhibition to the propagation of viruses [19,20]. Pre-injection of the recombinant proteins of ALFs could reduce the cumulative mortality of shrimp infected with *Vibrio harveyi* [21]. Silencing of ALFs could lead to susceptibility of shrimp to bacterial infection [22,23,24]. All these studies revealed that ALFs played important roles in crustacean innate immunity. 

Various isoforms of ALFs co-existing in one species exhibited different antimicrobial activities. Seven ALF isoforms were identified in the Chinese shrimp *Fenneropenaeus chinensis* [25] and five ALF isoforms were reported in the oriental river prawn *Macrobrachium nipponense* [26]. Discovery of more ALFs is important for the development of AMP drugs and disease control strategies in aquaculture. In our previous study, an ALF (*EcALF1*) gene was identified and characterized in the ridgetail prawn *Exopalaemon carinicauda*, which showed both antibacterial and antiviral activities [27]. In order to learn the diversity and function of ALFs in the ridgetail prawn, other ALF isoforms were isolated from a transcriptome data and their functions were analyzed. The present data will provide new clues for understanding the role of ALFs in crustacean immunity. 

## 2. Results

### 2.1. Sequences Characters and Phylogenic Analysis of EcALFs

**Figure 1:**
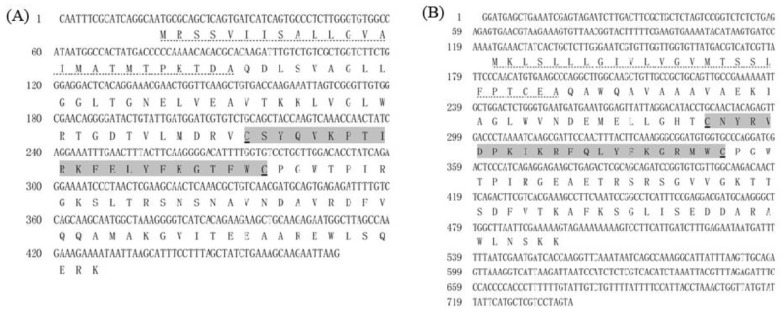

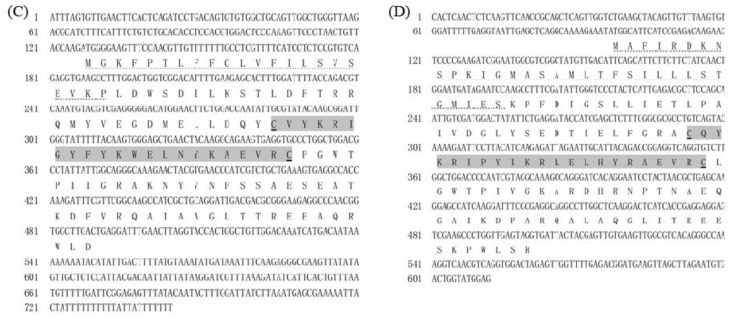
Nucleotide and amino acid sequences of EcALFs. (**A**) Showed the sequences of EcALF2; (**B**) showed the sequences of EcALF3; (**C**) showed the sequences of EcALF4; (**D**) showed the sequences of EcALF5. Predicted signal peptide was dotted underlined. The putative LPS-binding domain was marked with gray background and conserved cysteine residues were underlined.

**Figure 2:**
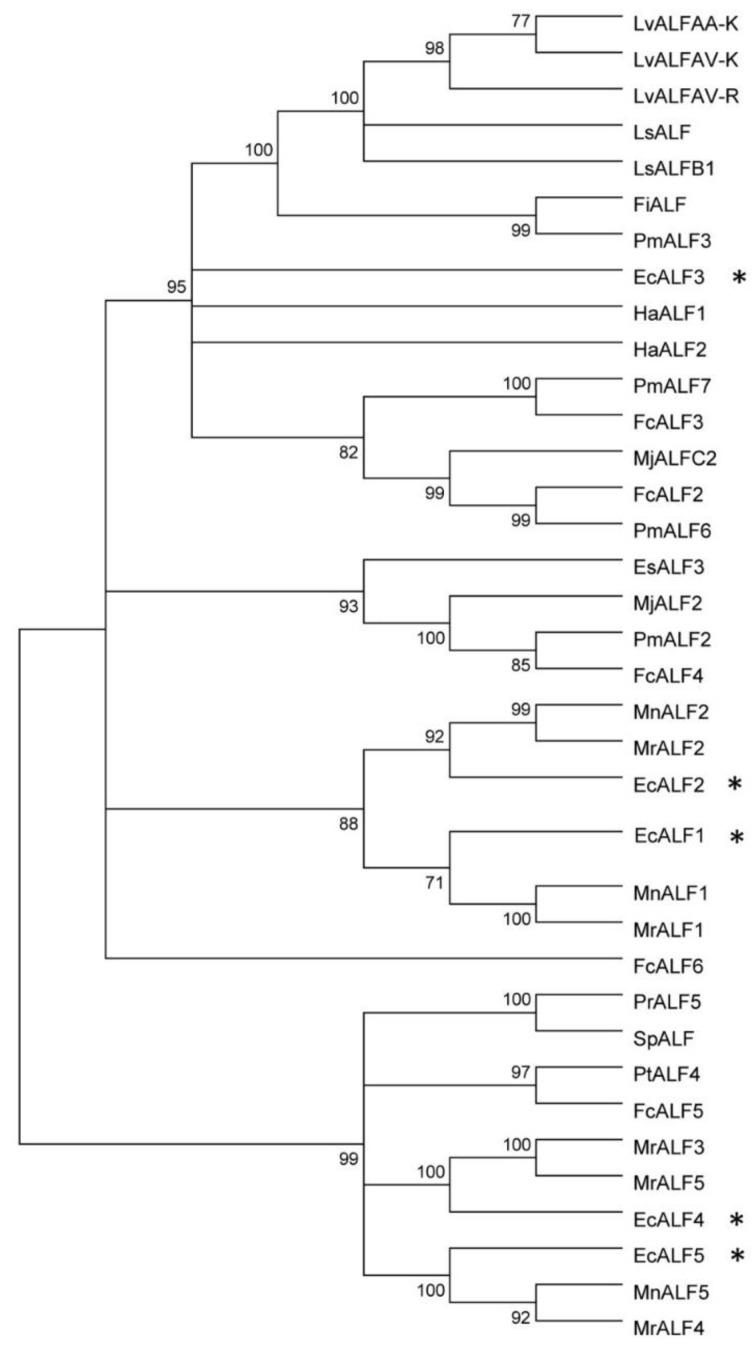
Bootstrapping phylogenetic analysis of ALFs from *E. carinicauda* and other crustaceans. The information of used ALF genes was listed in Supplementary Table S1. Whole deduced amino acid sequences except signal peptides of all genes were used for phylogenic analysis with neighbor-joining method. Bootstrap value was set at 1000. Percentage of bootstrap replications was shown in the figure. All the isoforms of EcALFs in the phylogenetic tree were labeled with stars (*).

Four full length cDNA sequences of *EcALFs* were confirmed by PCR and Sanger sequencing. As shown in Figure 1, their deduced amino acid sequences contained a signal peptide and a putative LPS-binding domain (LBD). Two conserved cysteine residues were identified in each predicted LBD. A phylogenetic tree was constructed by neighbor-joining method to analyze the evolutional relationship among *EcALFs* and other ALF sequences in crustaceans. As shown in Figure 2, *EcALF2* and the previously reported *EcALF1* were clustered into one group, which also included *MnALF2* and *MrALF2*. *EcALF3* was clustered together with *HaALF1* and *HaALF2*. *EcALF4* and *EcALF5* were classified into one group, which had a closer relationship with *MrALF3*, *MrALF5*, *MnALF5*, and *MrALF4*. 

### 2.2. Tissue Distribution of Different EcALFs Transcripts

**Figure 3:**
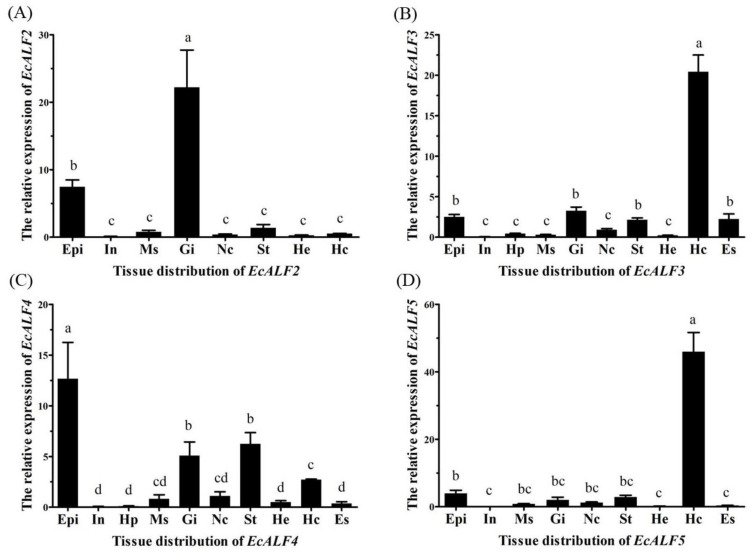
Tissue distribution of *EcALF2* (**A**); *EcALF3* (**B**); *EcALF4* (**C**) and *EcALF5* (**D**). Epi, epidermis; In, intestine; Hp, hepatopancreas; Ms, muscle; Gi, gill; Nc, nerve cord; St, stomach; He, heart; Hc, hemocytes; Es, eyestalk. Different lowercase letters (a, b, bc, c, cd, and d) showed significant differences of the expression levels of each EcALF gene among diverse tissues at *p* < 0.01.

The expression levels of *EcALFs* were analyzed in ten tissues of the prawn. *EcALF2* showed the highest expression level in the gill, relatively high expression level in the epidermis and low expression level in the stomach, muscle, hemocytes, nerve cord, and heart, while there was no expression in intestine, hepatopancreas, and eyestalk (Figure 3A). *EcALF3* transcripts were detected in all tested tissues, and the highest expression level was observed in hemocytes (Figure 3B). *EcALF4* was mainly expressed in epidermis, followed by stomach, gill, and hemocytes (Figure 3C). *EcALF5* was prominently expressed in hemocytes, whereas very low expression was detected in other tissues (Figure 3D). Different *EcALFs* exhibited various tissue expression profiles.

### 2.3. Minimal Inhibitory Concentrations (MIC) of the Synthetic EcLBD Peptides

In order to examine the antimicrobial activities of EcALFs, EcLBD peptides corresponding the LBD of EcALFs were synthesized based on their sequence information (Table 1). The MIC values of each synthetic EcLBD are shown in Table 2. The EcLBD2 peptide could inhibit the growth of *V. harveyi* with an MIC range of 32–64 μM. The EcLBD3 peptide showed activities against *V. harveyi*, *Photobacterium damselae*, and *Kocuria rhizophila* with the MIC ranges of 8–16, 32–64, and 32–64 μM, respectively. The MIC ranges of EcLBD4 peptide for bacteria *Vibrio alginolyticus* and *Staphylococcus epidermidis* were 16–32 μM and 32–64 μM. The EcLBD5 peptide showed an activity against *V. alginolyticus* with the MIC range of 16–32 μM. 

### 2.4. Antiviral Activity of EcLBD Peptides

**Figure 4:**
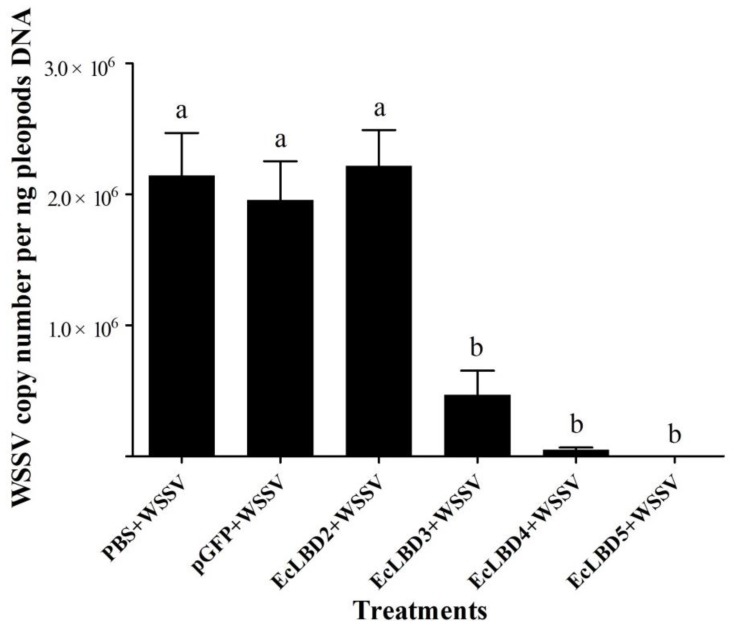
Detection of viral load in *E. carinicauda* after infection of WSSV pre-incubated with EcALFs-LBD. ‘EcLBD2 + WSSV’, ‘EcLBD3 + WSSV’, ‘EcLBD4 + WSSV’, and ‘EcLBD5 + WSSV’ were groups injected with 64 μM EcLBD2, EcLBD3, EcLBD4 and EcLBD5 peptide pre-incubated WSSV, respectively. ‘PBS + WSSV’ was group injected with WSSV, and ‘pGFP + WSSV’ was group injected with pGFP pre-incubated WSSV as positive controls. The copy number of WSSV was shown by measuring the expression level of VP28. Different lowercase letters (a and b) showed significant differences of copy number of WSSV between different groups at *p* < 0.01.

The antiviral activities of different EcLBD peptides against white spot syndrome virus (WSSV) were detected. As shown in Figure 4, EcLBD peptides exhibited different antiviral activities against WSSV at 48 h after injection. The WSSV copy numbers in the prawn from the PBS + WSSV group and pGFP + WSSV group were 2.16 × 10^6^ copies/ng DNA and 2.14 × 10^6^ copies/ng DNA, respectively. The WSSV copy numbers in the prawn from EcLBD3 + WSSV group, EcLBD4 + WSSV group, and EcLBD5 + WSSV group were 1.06 × 10^6^ copies/ng DNA, 1.47 × 10^5^ copies/ng DNA and 4.18 × 10^2^ copies/ng DNA, respectively, which were much lower than those in pGFP + WSSV group and PBS + WSSV group. The WSSV copy number in the prawn from EcLBD2 + WSSV group showed no significant difference with those in control groups.

### 2.5. Optimization of dsRNA Dosage for Different EcALFs in E. carinicauda

**Figure 5:**
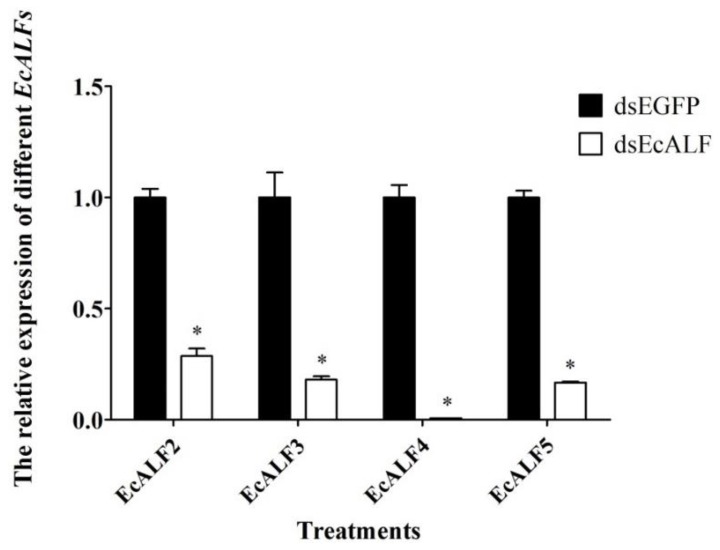
The silencing efficiency of different *EcALFs*. Eight microgram dsRNA of each *EcALF* gene was injected into one individual. Significant differences of the expression levels of each *EcALF* gene between treatment group (*EcALF* dsRNA injection group) and control group (EGFP dsRNA injection group) were labeled with a star (*) at *p* < 0.01.

To uncover the immune function of EcALFs in vivo, the RNA interference assay was carried out for *EcALFs* silencing. Firstly, the dosage of dsRNA for each *EcALF* was optimized by injecting 1, 4, and 8 μg dsRNA to each animal. After dosage optimization, 8 μg dsRNA for each *EcALF* was chosen for further RNAi experiments. At this dosage, the expression levels of *EcALF2*, *EcALF3*, *EcALF4*, and *EcALF5* were reduced by 67, 79, 97, and 83%, respectively (Figure 5). 

### 2.6. Effects of EcALFs Silencing on Bacterial Proliferation in the Hepatopancreas of the Prawn

**Figure 6:**
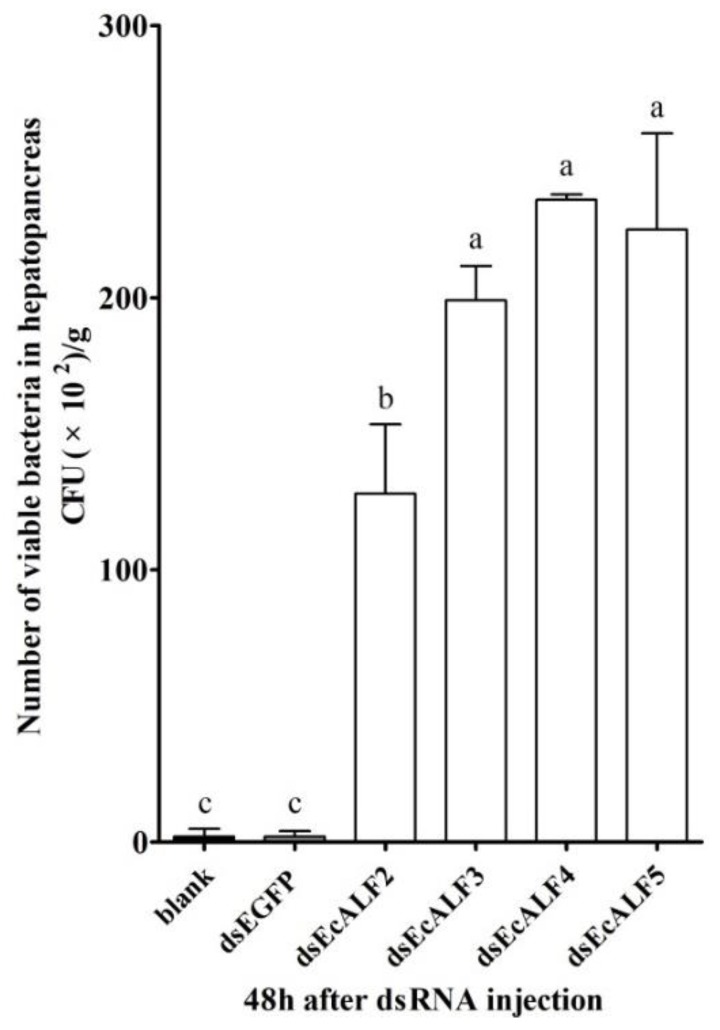
The total viable bacteria counts in hepatopancreas of EcALFs-silenced prawn. The data were obtained from three independent repeats with three individuals in each sample. Lowercase letters (a, b and c) showed significant differences of the number of total viable bacteria among different groups at *p* < 0.01.

The viable bacteria in the hepatopancreas of the prawn were analyzed to detect the function of different EcALFs. At 48 h after different EcALFs dsRNA injection, the hepatopancreas of prawn was collected, and the amount of total viable bacteria cultured on thiosulfate-citrate-bile salts-sucrose (TCBS) agar was counted. The colony number of TCBS cultured bacteria in the hepatopancreas of the prawn after silencing with different EcALFs dsRNA, including EcALF2-EcALF5, was about 1.28 × 10^4^ CFU/g, 1.99 × 10^4^ CFU/g, 2.36 × 10^4^ CFU/g, and 2.25 × 10^4^ CFU/g, respectively, which was 42.7-, 66-, 78.6-, and 75-fold higher than that in the prawn injected with EGFP dsRNA (Figure 6).

In order to identify the dominant bacteria in the hepatopancreas of prawn, the 16S rRNA sequences of dominant and representative colonies were sequenced. In EcALF2-silenced prawn, the dominant bacteria were *P. damselae* and *Vibrio xuii*, and the amounts of two strains were 1.7 × 10^3^ CFU/g and 7.4 × 10^3^ CFU/g. In EcALF3 and EcALF5-silenced prawn, the dominant strain was *V. alginolyticus*, and the amounts were 1.33 × 10^4^ CFU/g and 1.83 × 10^4^ CFU/g, respectively. In EcALF4-silenced prawn, the dominant bacteria were *V. harveyi* and *Vibrio parahaemolyticus*, and the amounts of two strains were 1.19 × 10^4^ CFU/g and 1.17 × 10^4^ CFU/g, respectively. 

### 2.7. Hemolytic Property of EcLBD Peptides

**Figure 7:**
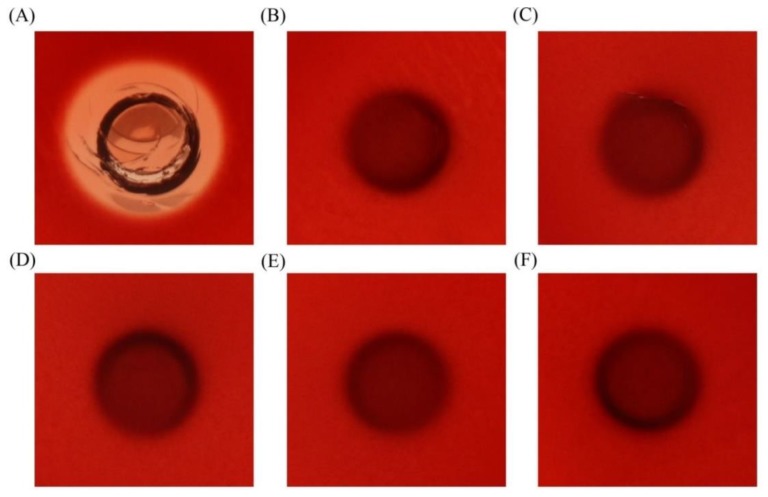
The hemolytic phenotypes of EcLBD2 (**C**); EcLBD3 (**D**); EcLBD4 (**E**); and EcLBD5 (**F**) on sheep blood agar. The same volume of 0.2% Triton X-100 (**A**) and the same amount of pGFP peptide (**B**) was used as positive and negative controls.

To detect the hemolytic property of EcLBD peptides, hemolysis test was performed using blood agar plates. All the EcLBD peptides showed no obvious hemolytic activity (Figure 7). 

## 3. Discussions

In the present study, four *EcALF* isoforms were identified from the ridgetail prawn *E. carinicauda*. Tissue distribution profiles of *EcALFs* were related to their biological functions. *EcALF2* and *EcALF4* were mainly distributed in gill, epidermis, and stomach, while *EcALF3* and *EcALF5* were mainly in hemocytes. The previously reported *EcALF1* was also highly expressed in hemocytes and gill [27]. Gill, epidermis, and stomach, which directly contact with environmental microbes, constitute the first physical barrier of crustacean immune system. Hemocytes are important components in crustacean innate immunity with the function for recognizing pathogens and phagocytosis [28]. The high expression level of *EcALFs* in these tissues indicated the important roles of *EcALFs* in defending the invasion of pathogens in *E. carinicauda*.

As a kind of antimicrobial peptides, ALFs usually show a broad-spectrum of antimicrobial activities against bacteria, viruses and fungi [23,25,26]. Since the LPS-binding domain (LBD) of ALF exerts the major antimicrobial activity, the use of synthetic LBD peptides in analyzing antimicrobial activities of ALFs has become a common method in ALFs researches [20,29,30]. The synthetic EcLBD1 peptide exhibited strong activities against several Gram-negative bacteria and Gram-positive bacteria [27], while the LBDs from other EcALFs presented in this study showed distinct antibacterial activities. The EcLBD3 and EcLBD4 had broad-spectrum activities against Gram-negative and Gram-positive bacteria, while EcLBD2 and EcLBD5 only showed activity against Gram-negative bacteria. After incubation with WSSV, all EcLBDs except EcLBD2 could inhibit the in vivo viral multiplication. The diverse antimicrobial activities of different EcALFs indicated that they might play a synergistic effect in the immune system. Therefore, antimicrobial agents against different pathogens could be developed according to the diverse activities of distinct ALFs.

RNA interference analysis showed that ALFs could affect the in vivo bacteria balance of animals. After silencing of different *EcALFs* with dsRNA, the prawn became unhealthy with a lesion in hepatopancreas. The *EcALFs*-silenced prawn showed typical symptoms with empty stomach and midgut, atrophied and whitish hepatopancreas. This result was very similar to the previous report about *EcALF1* [27]. Silencing of *EcALFs* finally resulted in moribund state of the prawn, which was also reported in *Penaeus monodon*, in which the silencing of *ALFPm3* led to rapid mortality of the shrimp [22]. Bacteria identification in the hepatopancreas of *EcALFs*-silenced prawn showed a rapid increase of the number of pathogenic *Vibrio*. *Vibrio* such as *V. harveyi*, *V. parahaemolyticus*, *V. alginolyticus*, *Vibrio anguillarum*, *Vibrio vulnificus*, were the most common and serious pathogens in fish and shellfish [31]. Among them, *V. parahaemolyticus* was regarded as the major pathogen for the severe shrimp disease named acute hepatopancreatic necrosis disease (AHPND), which was characterized by empty stomach and midgut, and atrophied and whitish hepatopancreas [32]. The phenomenon caused by *EcALFs* silencing indicated that *EcALFs* played important roles in modulating the in vivo flora balance. Silencing of *EcALFs* might disrupt the balance and caused the pathogenic bacterial proliferation in hepatopancreas and the morbidity of prawn. 

Diversity and broad spectrum antimicrobial activity are notable features of the ALF antimicrobial peptides. From the alignment result (Figure 2), we found that EcALFs were clustered into three categories. In penaeid shrimp, ALF peptides were classified into four groups [33]. These data indicated that ALFs from one species or one family showed great sequence differences. The diversity of the sequences led to the differences of their activities. Therefore, the information might provide important clues for drug development. For further application, many characteristics of the peptides—such as stability, cytotoxicity, and biodisponibility problems—should be investigated [34]. Previous studies revealed that a modified LBD peptide LBDv had good thermal stability and low cytotoxicity against Sf9 cells and crayfish hemocytes [35]. The present study also showed that EcLBD peptides did not have obvious hemolytic activity. These features enable LBD peptides of ALFs to be appropriate candidates for drug development.

In summary, the present study identified and characterized four *EcALF* isoforms from the prawn *E. carinicauda*. High expression levels of *EcALFs* were all detected in immune related tissues. Different EcALFs exhibited diverse antibacterial and antiviral activities. The gene silencing of *EcALFs* resulted in pathogenic bacterial proliferation in hepatopancreas of the prawn. The data reveals the important functions of *EcALFs* in innate immunity of *E. carinicauda* and will provide new clues for developing antimicrobial agents.

## 4. Materials and Methods

### 4.1. Animals and Tissue Sampling

Healthy prawn *E. carinicauda*, with an average body length of 4.15 ± 0.65 cm and body weight of 0.93 ± 0.33 g, were acclimatized in the aerated seawater at 25 °C for one day before experiments. Hemolymph was collected from 20 individuals and centrifuged at 800× *g*, 4 °C for 10 min. Different tissues including eyestalk, epidermis, gill, heart, hepatopancreas, intestine, muscle, nerve cord, and stomach from 10 individuals were collected and preserved in liquid nitrogen for expression analysis of target genes. 

### 4.2. Sequence Analysis of EcALFs

The cDNA sequences of *EcALFs* were obtained from a transcriptome library of the ridgetail prawn *Exopalaemon carinicauda*. PCR and Sanger sequencing were performed to confirm the open reading frame (ORF) of *EcALFs*, and the primers for PCR amplification were listed in Supplementary Table S1. The nucleotide sequences and deduced amino acid sequences of *EcALFs* were analyzed with the BLAST algorithm. CBS prediction server (http://www.cbs.dtu.dk/services) was used to predict the signal peptides of EcALFs. The amino acid sequences of various ALFs (Supplementary Table S2) were obtained from the NCBI database (NCBI, http://www.ncbi.nlm.nih.gov/BLAST/). Multiple sequence alignment was performed by ClustalW2 program, and a phylogenetic tree was constructed with MEGA 6.0 software.

### 4.3. Total RNA Extraction and RT-qPCR Analysis

The total RNA from different tissues was extracted with RNAiso Plus reagent (TaKaRa, Dalian, China). The cDNA was synthesized from 1 μg total RNA using PrimeScript RT Reagent Kit (TaKaRa, Dalian, China), following the manufacturer’s instructions.

The expression levels of *EcALFs* in various tissues were measured by quantitative real-time PCR (qPCR). The primers used for qPCR were presented in Supplementary Table S1, and the program of qPCR was set as follows: 95 °C for 15 min, followed by 40 cycles of 95 °C for 15 s, at the annealing temperature for 15 s and 72 °C for 20 s. The specificity of PCR product was validated by melting-curve analysis which was performed at the end of each PCR reaction. The primers 18S-F and 18S-R (Supplementary Table S1) were designed to detect the expression of the reference gene, 18S rRNA.

### 4.4. Synthesis of EcLBD Peptides

The peptides designed based on the sequences of the LBD of different EcALFs were synthesized by a commercial company (Shanghai Ziyu Biotechnology Co., Ltd., Shanghai, China). A peptide corresponding to a fragment of green fluorescent protein (pGFP) was also synthesized as a negative control. The amino acid sequences of these peptides were present in Table 1. Fmoc strategy was used for peptide synthesis. Briefly, one gram Rink Amide (RAM) Resin was added into a reaction column and swelled for 30 min with dichloromethane (DCM). The Fmoc was de-protected by 20% Piperidine/Dimethyl formamide (DMF) for 10 min, and then washed by DMF. 0.9 mmol Fmoc-Pro-OH, 0.9 mmol HOBT, 0.9 mmol HBTU, and 0.9 mmol DIEA were added into the resin for 40 min. Then resins were washed two times by DMF. The length of the peptide chain was increased by repeating the steps de-protection, coupling, and washing until all the amino acids were sequentially coupled to the chain. The peptide was finally de-protected after the last amino acid was coupled into the chain. Then the resins were washed with MeOH for three times. Then the dried resins were added with appropriate amount of cleavage solution and incubated at 40 °C for 3.5 h. The reaction solution was then filtered and the synthesized peptide was precipitated by adding ether into the solution. Then the peptide was air-dried and lyophilized. The synthesized peptide was dissolved in pure water and reacted with hydrogen peroxide for 10 min for disulfide bond formation. Then the synthesized peptide was purified by high performance liquid chromatograph (HPLC), and confirmed by mass spectrometry. The information of HPLC, mass spectrometry, and the degree of purity and mass values were shown in Supplementary Tables S3–S5.

### 4.5. Minimal Inhibitory Concentration (MIC) Assay

MIC assay was carried out to examine the antibacterial activities of EcLBDs following the method described previously [26]. Briefly, various bacteria strains were incubated in an appropriate medium overnight at an optimum temperature. *Kocuria rhizophila*, *Staphylococcus epidermidis*, *Bacillus licheniformis*, *Vibrio parahaemolyticus*, and *Escherichia coli* were incubated in Lysogeny broth (LB) medium at 37 °C. *Vibrio alginolyticus*, *Vibrio harveyi*, and *Photobacterium damselae* were incubated in Tryptic Soy Broth (TSB) medium at 28 °C. Then the cultured bacteria were transferred to fresh fluid medium and cultured for 6 h before use. The procedures for MIC assay were the same as described by Yang et al. [32]. In brief, 133 μL fluid medium, 15 μL peptide solution, and 2 μL bacteria culture in the medium were added in each well of the 96-well plates. Different medium were added according to the bacteria strains. The finally serial concentration of EcLBD peptides were 64, 32, 16, 8, 4, 2, and 1 μM. PBS solution and the same concentration of pGFP peptide were set as blank group and negative control, respectively. The 96-well plates were incubated for 6–8 h at the optimum temperature depending on different bacteria strains. The absorbance at 600 nm and 560 nm were measured to estimate the amount of Gram-positive and Gram-negative bacteria by a precision micro-plate reader (TECAN infinite M200 PRO, Salzburg, Austria). The assay was performed in triplicates.

### 4.6. Antiviral Activity of EcLBDs

For detection on the antiviral activity of the synthetic peptides, 500 copies/μL WSSV was incubated with 64 μM synthetic peptides or PBS for 2 h before injection. WSSV used in this experiment was obtained from pathologically infected shrimp, and the virus extraction method was the same as previous description [36]. For the injection experiment, 120 individuals were divided into six groups including EcLBD2 + WSSV, EcLBD3 + WSSV, EcLBD4 + WSSV, EcLBD5 + WSSV, pGFP + WSSV, and PBS + WSSV. In each group, 10 μL of the above mixture was injected into each individual. At 24 h and 48 h after injection, the pleopods of prawn were collected and the pleopods of three individuals were put together as one sample. Five replicates were prepared for each group at one time point. Then, total DNA of each sample was extracted with the Genomic DNA Kit (Tiangen, Beijing, China). The viral copy number was detected by real-time PCR with WSSV VP28 specific primers (Supplementary Table S1) as described by Sun et al. [36]. The standard curve was constructed with the dilution of the plasmid DNA containing a fragment of VP28 gene from WSSV.

### 4.7. DsRNA Synthesis and Gene Silencing of EcALFs

Double stranded RNA (dsRNA) of each EcALFs was synthesized to perform RNA interference. The EGFP dsRNA, based on a fragment of the EGFP gene, was also synthesized as a negative control. The DNA templates for dsRNA synthesis were amplified from the cDNA sequence EcALF of E. carinicauda with specific primers (Table S1), and a 289 bp DNA fragment of the EGFP gene was also amplified with dsEGFP-F and dsEGFP-R primers (Table S1). DsRNA was synthesized with TranscriptAid T7 High Yield Transcription Kit (Thermo Fisher Scientific, Waltham, MA, USA), following the manufacturers’ protocols. To detect the optimal silencing dose of EcALFs dsRNA, 150 individuals were separated into five groups including dsEcALF2, dsEcALF3, dsEcALF4, dsEcALF5, and dsEGFP. Each group was divided into three sub-groups with 10 individuals. In different sub-group, each shrimp was injected with 1, 4, and 8 μg dsRNA, respectively. The total RNA from cephalothoraxes of four prawns in each sub-group was extracted at 48 h after dsRNA injection. The RT-qPCR was performed to analyze the RNAi efficiency of each EcALF. 4.8. Bacterial Count and Strain Identification in EcALFs-Silenced Prawn

Five groups (four dsEcALFs groups and one dsEGFP group) were set in this experiment, and each group that contained 30 individuals was divided into three replicated sub-groups. Each individual was injected with 8 μg *EcALFs* dsRNA or EGFP dsRNA. The hepatopancreas was collected at 48 h after dsRNA injection, and was crushed in sterile phosphate buffered saline (PBS). Then, 100 μL tissue homogenate was seeded onto TCBS agar media and cultured at 28 °C for 18 h. The number of total viable bacteria was counted and the representative single colonies were cultured overnight in TSB + 2% NaCl liquid media. 100 µL bacteria suspension was centrifuged at 10,000 rpm to remove the media. Then, 100 μL sterile water was added to suspend the bacteria. The bacteria was boiled for 10 min and centrifuged to collect the supernatant which was used as genomic DNA templates. The 16S rRNA partial sequence of each colony was amplified by PCR with universal primers 27F and 1492R (Supplementary Table S1). The PCR program was as follows: denaturation at 95 °C for 2 min, 35 cycles of 94 °C for 45 s, 55 °C for 30 s, and 72 °C for 100 s; with a final extension at 72 °C for 10 min. The PCR products were sequenced by Sangon Biotechnology Company Ltd. (Shanghai, China).

### 4.8. Hemolytic Activity of EcLBDs

The hemolytic property of EcLBD2, EcLBD3, EcLBD4, and EcLBD5 was detected using sheep blood agar plates (Qingdao Hope Bio. Technology Co., Ltd., Qingdao, China). The Oxford cup was placed on the surface of blood agar plate, and each one was added with 60 μL peptides (64 μM). The same amount of pGFP peptide was tested as a negative control, and the same volume of 0.2% Triton X-100 was set as a positive control. Then the plates were incubated at 30 °C for 8 h. 

### 4.9. Statistical Analyses

The statistical significance between treatments and controls and different treatments was analyzed using SPSS statistics 20 software. The analysis was performed by variance (ANOVA) and Duncan’s Multiple Comparisons. The significant differences at *p* < 0.01 were labeled with lowercase letters or star.

## Figures and Tables

**Table 1:** Sequence information of synthetic peptides

EcLBD2	Ac-V(CSYQVKPTIRKFELYFKGTFWC)P-NH_2_
EcLBD3	Ac-T(CNYRVDPKIKRFQLYFKGRMWC)P-NH_2_
EcLBD4	Ac-Y(CVYKRIGYFYKWELNYKAEVRC)P-NH_2_
EcLBD5	Ac-A(CQYKRIPYIKRLELHYRAEVRC)L-NH_2_
pGFP	Ac-TTGKLPVPWPTLVTTFSYGVQCFS-NH_2_

Note: ‘Ac-’ represents acetylation of the *N*-terminal amino acid residue; ‘-NH_2_’ represents amidation of the *C*-terminal amino acid residue; parentheses at two sides of cysteine amino acids show a disulfide bond. The peptides were designed based on the sequences of the LBD of different EcALFs and a fragment of EGFP amino acid sequence.

**Table 2:** Minimal inhibitory concentration of synthetic EcLBDs peptides on different bacteria

Bacteria	MIC (μM)			
	EcLBD2	EcLBD3	EcLBD4	EcLBD5
Gram-positive bacteria				
*K. rhizophila* ATCC 9341	>64	32–64	>64	>64
*S. epidermidis* ATCC 12228	>64	>64	32–64	>64
*B. licheniformis* ATCC 11946	>64	>64	>64	>64
Gram-negative bacteria				
*V. alginolyticus* ATCC 17749	>64	>64	16–32	16–32
*V. harveyi* ATCC 33842	32–64	8–16	>64	>64
*P. damselae* ATCC 33539	>64	32–64	>64	>64
*E. coli* ATCC 25922	>64	>64	>64	>64
*V. parahaemolyticus* ATCC 17802	>64	>64	>64	>64

## Supplementary Materials

The following are available online at http://www.mdpi.com/1660-3397/16/5/145/s1, Table S1: Primer sequences and corresponding annealing temperature of genes, Table S2: Protein information of ALFs used in the present study, Table S3: Information of HPLC analysis, Table S4: Information of mass analysis, Table S5: Purity and mass values of EcLBD peptides.

## References

[1] Steiner H., Hultmark D., Engstrom A., Bennich H., Boman H.G. (1981). Sequence and specificity of 2 anti-bacterial proteins involved in insect immunity. Nature.

[2] Martin L., van Meegern A., Doemming S., Schuerholz T. (2015). Antimicrobial peptides in human sepsis. Front. Immunol..

[3] Tam J.P., Wang S., Wong K.H., Tan W.L. (2015). Antimicrobial peptides from plants. Pharmaceuticals.

[4] Yi H.Y., Chowdhury M., Huang Y.D., Yu X.Q. (2014). Insect antimicrobial peptides and their applications. Appl. Microbiol. Biotechnol..

[5] Rosa R.D., Barracco M.A. (2010). Antimicrobial peptides in crustaceans. Invert. Surviv. J..

[6] Tassanakajon A., Amparyup P., Somboonwiwat K., Supungul P. (2011). Cationic antimicrobial peptides in penaeid shrimp. Mar. Biotechnol..

[7] Hoess A., Watson S., Siber G.R., Liddington R. (1993). Crystal structure of an endotoxin-neutralizing protein from the horseshoe crab, Limulus anti-LPS factor, at 1.5 A resolution. EMBO J..

[8] Hancock G.E., Speelman D.J., Heers K., Bortell E., Smith J., Cosco C. (1996). Generation of atypical pulmonary inflammatory responses in BALB/c mice after immunization with the native attachment (G) glycoprotein of respiratory syncytial virus. J. Virol..

[9] Andra J., Lamata M., Martinez de Tejada G., Bartels R., Koch M.H., Brandenburg K. (2004). Cyclic antimicrobial peptides based on Limulus anti-lipopolysaccharide factor for neutralization of lipopolysaccharide. Biochem. Pharmacol..

[10] Tanaka S., Nakamura T., Morita T., Iwanaga S. (1982). Limulus anti-LPS factor: An anticoagulant which inhibits the endotoxin-mediated activation of Limulus coagulation system. Biochem. Biophys. Res. Commun..

[11] Morita T., Ohtsubo S., Nakamura T., Tanaka S., Iwanaga S., Ohashi K., Niwa M. (1985). Isolation and biological activities of Limulus anticoagulant (anti-LPS factor) which interacts with lipopolysaccharide (LPS). J. Biochem..

[12] Muta T., Miyata T., Tokunaga F., Nakamura T., Iwanaga S. (1987). Primary structure of antilipopolysaccharide factor from American horseshoe crab, *Limulus polyphemus*. J. Biochem..

[13] Beale K.M., Towle D.W., Jayasundara N., Smith C.M., Shields J.D., Small H.J., Greenwood S.J. (2008). Anti-lipopolysaccharide factors in the American lobster *Homarus americanus*: Molecular characterization and transcriptional response to Vibrio fluvialis challenge. Comp. Biochem. Phys. D.

[14] Imjongjirak C., Amparyup P., Tassanakajon A., Sittipraneed S. (2007). Antilipopolysaccharide factor (ALF) of mud crab *Scylla paramamosain*: Molecular cloning, genomic organization and the antimicrobial activity of its synthetic LPS binding domain. Mol. Immunol..

[15] Nagoshi H., Inagawa H., Morii K., Harada H., Kohchi C., Nishizawa T., Taniguchi Y., Uenobe M., Honda T., Kondoh M. (2006). Cloning and characterization of a LPS-regulatory gene having an LPS binding domain in kuruma prawn *Marsupenaeus japonicus*. Mol. Immunol..

[16] Somboonwiwat K., Marcos M., Tassanakajon A., Klinbunga S., Aumelas A., Romestand B., Gueguen Y., Boze H., Moulin G., Bachere E. (2005). Recombinant expression and anti-microbial activity of anti-lipopolysaccharide factor (ALF) from the black tiger shrimp *Penaeus monodon*. Dev. Comp. Immunol..

[17] Prapavorarat A., Pongsomboon S., Tassanakajon A. (2010). Identification of genes expressed in response to yellow head virus infection in the black tiger shrimp, *Penaeus monodon*, by suppression subtractive hybridization. Dev. Comp. Immunol..

[18] Soonthornchai W., Rungrassamee W., Karoonuthaisiri N., Jarayabhand P., Klinbunga S., Soderhall K., Jiravanichpaisal P. (2010). Expression of immune-related genes in the digestive organ of shrimp, *Penaeus monodon*, after an oral infection by *Vibrio harveyi*. Dev. Comp. Immunol..

[19] Tharntada S., Ponprateep S., Somboonwiwat K., Liu H., Soderhall I., Soderhall K., Tassanakajon A. (2009). Role of anti-lipopolysaccharide factor from the black tiger shrimp, *Penaeus monodon*, in protection from white spot syndrome virus infection. J. Gen. Virol..

[20] Li S., Guo S., Li F., Xiang J. (2014). Characterization and function analysis of an anti-lipopolysaccharide factor (ALF) from the Chinese shrimp *Fenneropenaeus chinensis*. Dev. Comp. Immunol..

[21] Ponprateep S., Somboonwiwat K., Tassanakajon A. (2009). Recombinant anti-lipopolysaccharide factor isoform 3 and the prevention of vibriosis in the black tiger shrimp, *Penaeus monodon*. Aquaculture.

[22] Ponprateep S., Tharntada S., Somboonwiwat K., Tassanakajon A. (2012). Gene silencing reveals a crucial role for anti-lipopolysaccharide factors from *Penaeus monodon* in the protection against microbial infections. Fish Shellfish Immunol..

[23] De la Vega E., O’Leary N.A., Shockey J.E., Robalino J., Payne C., Browdy C.L., Warr G.W., Gross P.S. (2008). Anti-lipopolysaccharide factor in *Litopenaeus vannamei* (LvALF): A broad spectrum antimicrobial peptide essential for shrimp immunity against bacterial and fungal infection. Mol. Immunol..

[24] Jiang H.S., Zhang Q., Zhao Y.R., Jia W.M., Zhao X.F., Wang J.X. (2015). A new group of anti-lipopolysaccharide factors from *Marsupenaeus japonicus* functions in antibacterial response. Dev. Comp. Immunol..

[25] Li S., Guo S., Li F., Xiang J. (2015). Functional diversity of anti-lipopolysaccharide factor isoforms in shrimp and their characters related to antiviral activity. Mar. Drugs.

[26] Wang G., Mishra B., Lau K., Lushnikova T., Golla R., Wang X. (2015). Antimicrobial peptides in 2014. Pharmaceuticals.

[27] Lv X., Li S., Liu F., Li F., Xiang J. (2017). Identification and function analysis of an anti-lipopolysaccharide factor from the ridgetail prawn *Exopalaemon carinicauda*. Dev. Comp. Immunol..

[28] Johansson M.W., Keyser P., Sritunyalucksana K., Soderhall K. (2000). Crustacean haemocytes and haematopoiesis. Aquaculture.

[29] Wang Y., Tang T., Gu J., Li X., Yang X., Gao X., Liu F., Wang J. (2015). Identification of five anti-lipopolysaccharide factors in oriental river prawn, *Macrobrachium nipponense*. Fish Shellfish Immunol..

[30] Imjongjirak C., Amparyup P., Tassanakajon A. (2011). Molecular cloning, genomic organization and antibacterial activity of a second isoform of antilipopolysaccharide factor (ALF) from the mud crab, *Scylla paramamosain*. Fish Shellfish Immunol..

[31] Chatterjee S., Haldar S. (2012). Vibrio related diseases in aquaculture and development of rapid and accurate identification methods. J. Mar. Sci. Res. Dev..

[32] Lightner D.V., Redman R.M., Pantoja C.R., Noble B.L., Tran L. (2012). Early mortality syndrome affects shrimp in Asia. Glob. Aquac. Adv..

[33] Rosa R.D., Vergnes A., de Lorgeril J., Goncalves P., Perazzolo L.M., Sauné L., Romestand B., Fievet J., Gueguen Y., Bachère E. (2013). Functional divergence in shrimp anti-lipopolysaccharide factors (ALFs): From recognition of cell wall components to antimicrobial activity. PLoS ONE.

[34] Boto A., de la Lastra J.M.P., González C.C. (2018). The road from host-defense peptides to a new generation of antimicrobial drugs. Molecules.

[35] Yang H., Li S., Li F., Xiang J. (2016). Structure and bioactivity of a modified peptide derived from the LPS-binding domain of an anti-lipopolysaccharide factor (ALF) of shrimp. Mar. Drugs.

[36] Sun Y.M., Li F.H., Xiang J.H. (2013). Analysis on the dynamic changes of the amount of WSSV in Chinese shrimp *Fenneropenaeus chinensis* during infection. Aquaculture.

