# Supplementary material for: Multiple Isoforms of Anti-Lipopolysaccharide Factors and Their Antimicrobial Functions in the Ridgetail Prawn Exopalaemon carinicauda
Source: Mar Drugs. 2018 Apr 27;16(5):145. doi: 10.3390/md16050145 (PMC5983276; doi:10.3390/md16050145)
Supplement: Supplementary file 1 [file marinedrugs-16-00145-s001.pdf]

Supplementary Tables

Supplementary Table 1 Primer sequences and corresponding annealing temperature of genes

Primer name	Primer sequence（5'-3'）	Annealing temperature (°C)
EcALF2F	CAATTTCGCATCAGGCAATGCG	60
EcALF2R	GCTTTCAGATAGCTAAAGGAAATG	
EcALF3F	GAATCTTGACTTCGCTGCTCTA	60
EcALF3R	CCTTTGGCTGATTATTTGAACC	
EcALF4F	ACGCATCTTTCATTTCTGTCTG	58
EcALF4R	TTGTCGTAATGGAGAGCAACTA	
EcALF5F	CAACTCTCAAGTTCAACCGCAG	60
EcALF5R	CCATACCAGTCACATTCTAAGC	
qEcALF2F	CCCAAAACAGACGCACAAGA	55
qEcALF2R	CCAACCAGGACACCAAAATG	
qEcALF3F	GCGTCCTTCAACCTGCTTCTT	53
qEcALF3R	CTGAGCCCCATAATCCAACAA	
qEcALF4F	TTGCCTCGTTTTCATCCTCT	52
qEcALF4R	TTCTTTGCCCTGCCAATAATAG	
qEcALF5F	AATGGCGTCGGCTATGTTGA	55
qEcALF5R	TGACCTCCGCTCTGTAATGC	
18S-F	TATACGCTAGTGGAGCTGGAA	55
18S-R	GGGGAGGTAGTGACGAAAAAT	
VP28-qF	AAACCTCCGCATTCCTGTGA	55
VP28-qR	TCCGCATCTTCTTCCTTCAT	
dsEcALF2F	TAATACGACTCACTATAGGGCAATTTCGCATCAGGCAATG	60
dsEcALF2R	TAATACGACTCACTATAGGGCTTAATTCTTGCTTTCAGAT	
dsEcALF3F	TAATACGACTCACTATAGGGGAGTGAACGTAAGAAAGTGT	60
dsEcALF3R	TAATACGACTCACTATAGGGATGCCTTTGGCTGATTATTT	
dsEcALF4F	TAATACGACTCACTATAGGGACGCATCTTTCATTTCTGTC	60
dsEcALF4R	TAATACGACTCACTATAGGGGTTCAAATCCTCAGTCAAGC	
dsEcALF5F	TAATACGACTCACTATAGGGATCCGAGACAAGAACTCCCC	60
dsEcALF5R	TAATACGACTCACTATAGGGTGTGACGCCAACTTCACAAC	
dsEGFP-F	TAATACGACTCACTATAGGGCAGTGCTTCAGCCGCTACCC	60
dsEGFP-R	TAATACGACTCACTATAGGGAGTTCACCTTGATGCCGTTCTT	
27F	AGAGTTTGATCMTGGCTCAG	55
1492R	TACGGYTACCTTGTTACGACTT	

Supplementary Table 2 Protein information of ALFs used in the present study

Gene name	Accession number	Species
MnALF1	ALF02818.1	*Macrobrachium nipponense*
MrALF1	AFW04304.1	*Macrobrachium rosenbergii*
MnALF2	ALF02817.1	*Macrobrachium nipponense*
MrALF2	ADI80707.1	*Macrobrachium rosenbergii*
HaALF2	ACC94269.1	*Homarus americanus*
MjALFC2	AME17862.1	*Marsupenaeus japonicus*
FcALF2	AFU61125.1	*Fenneropenaeus chinensis*
PmALF6	ADM21460.1	*Penaeus monodon*
PmALF7	ANP92039.2	*Penaeus monodon*
FcALF3	AFU61126.1	*Fenneropenaeus chinensis*
FcALF6	AFU61129.1	*Fenneropenaeus chinensis*
PmALF2	ABP73291.1	*Penaeus monodon*
FcALF4	AFU61127.1	*Fenneropenaeus chinensis*
FiALF	ADE27980.1	*Fenneropenaeus indicus*
PmALF3	ABP73289.1	*Penaeus monodon*
HaALF1	ACC94268.1	*Homarus americanus*
MjALF2	BAH22585.1	*Marsupenaeus japonicus*
MrALF3	ADI80708.1	*Macrobrachium rosenbergii*
MrALF5	AOF80307.1	*Macrobrachium rosenbergii*
MnALF5	ALF02821.1	*Macrobrachium nipponense*
MrALF4	AOF80306.1	*Macrobrachium rosenbergii*
PrALF5	AFA42333.1	*Portunus trituberculatus*
PtALF4	AFA42332.1	*Portunus trituberculatus*
FcALF5	AFU61128.1	*Fenneropenaeus chinensis*
SpALF	AHB62419.1	*Scylla paramamosain*
LsALF	ABJ90465.1	*Litopenaeus schmitti*
LsALFB1	AGH32549.1	*Litopenaeus stylirostris*
EsALF3	ADZ46233.1	*Eriocheir sinensis*
LvALFAV-R	ABB22832.1	*Litopenaeus vannamei*
LvALFAA-K	ABB22833.1	*Litopenaeus vannamei*
LvALFAV-K	ACT21197.1	*Litopenaeus vannamei*

Supplementary Table 3 Information of HPLC analysis

HPLCsystem	Agilent 1100
column	Gemini-NX 5μ C18 110A, 4.6*250mm
solvent gradient composition	solventA: 0.1% trifluoroacetic in 100% acetonitrile
	solventB: 0.1% trifluoroacetic in 100% water
	gradient: 0.01min: 25%A, 75%B; 25min: 50%A, 50%B; 25.1min: 100%A, 0%B; 30min: stop

Supplementary Table 4 Information of mass analysis

Instrument	Waters ZQ2000		
Probe:	ESI	Probe Bias:	＋4.5kv
Nebulizer Gas Flow:	1.5L/min	Detector:	1.5kv
CDL:	-20.0v	T. Flow:	0.2ml/min
CDL Temp:	250 °C	B. Conc:	50%H2O/50%ACN
Block Temp:	200 °C		

Supplementary Table 5 Purity and mass values of EcLBD peptides

Peptide Name	Purity	Molecular Weight
EcLBD2	97.63%	2980.57
EcLBD3	95.24%	3116.76
EcLBD4	95.33%	3131.7
EcLBD5	96.33%	3045.65
